# Hypothesis on energy storage by membrane proton buffers: Connections to sleep and memory

**DOI:** 10.1016/j.bbrep.2026.102857

**Published:** 2026-11-03

**Authors:** Alessandro Maria Morelli

**Affiliations:** University of Genoa, Italy

**Keywords:** Mitochondria, Chemiosmotic theory, Mitochondria derived vesicles, Memory, Sleep, Biological energy reserves

## Abstract

Extramitochondrial ATP synthesis appears widespread thanks to the formation of mitochondrial-derived vesicles (MDVs) that export the mitochondrial molecular machinery of oxidative phosphorylation (OXPHOS) to cellular and extracellular membrane systems, where OXPHOS can occur in two phases: first, protons are charged into membrane proton buffers, and then, "discharge" occurs with the coupled synthesis of ATP. The capacitance of membrane proton buffers increases in the presence of abundant basic proteins (such as the myelin sheath, which contains nearly 30% myelin basic protein) allowing the first phase of OXPHOS to optimally charge the proton buffer, which then allows for an efficient discharge phase with the synthesis of large amounts of ATP. This provides an energy storage function that was not predicted by the chemiosmotic theory. Since memory depends on the accumulation and expenditure of brain energy, possible connections between energy buffers and memory are being considered. This study reevaluates the experiments of Russian scientists who, half a century ago, achieved a biphasic oxidative phosphorylation with intermediate proton accumulation by an artificial hydrophobic proton buffer embedded in the lipid phase.

## Highlights

• ATP can be synthesized via extramitochondrial oxidative phosphorylation (OXPHOS).
• The chemiosmotic theory requires some modifications to be applicable outside the mitochondria.
• The protons move within the membrane traveling on the groups of the “heads” of phospholipids.
• Protons can be stored in basic proteins such as Myelin Basic Protein for brain energy storage.
• Proteins like Myelin Basic Protein help store proton and energy during sleep.

## Graphical abstract

**Figure:**
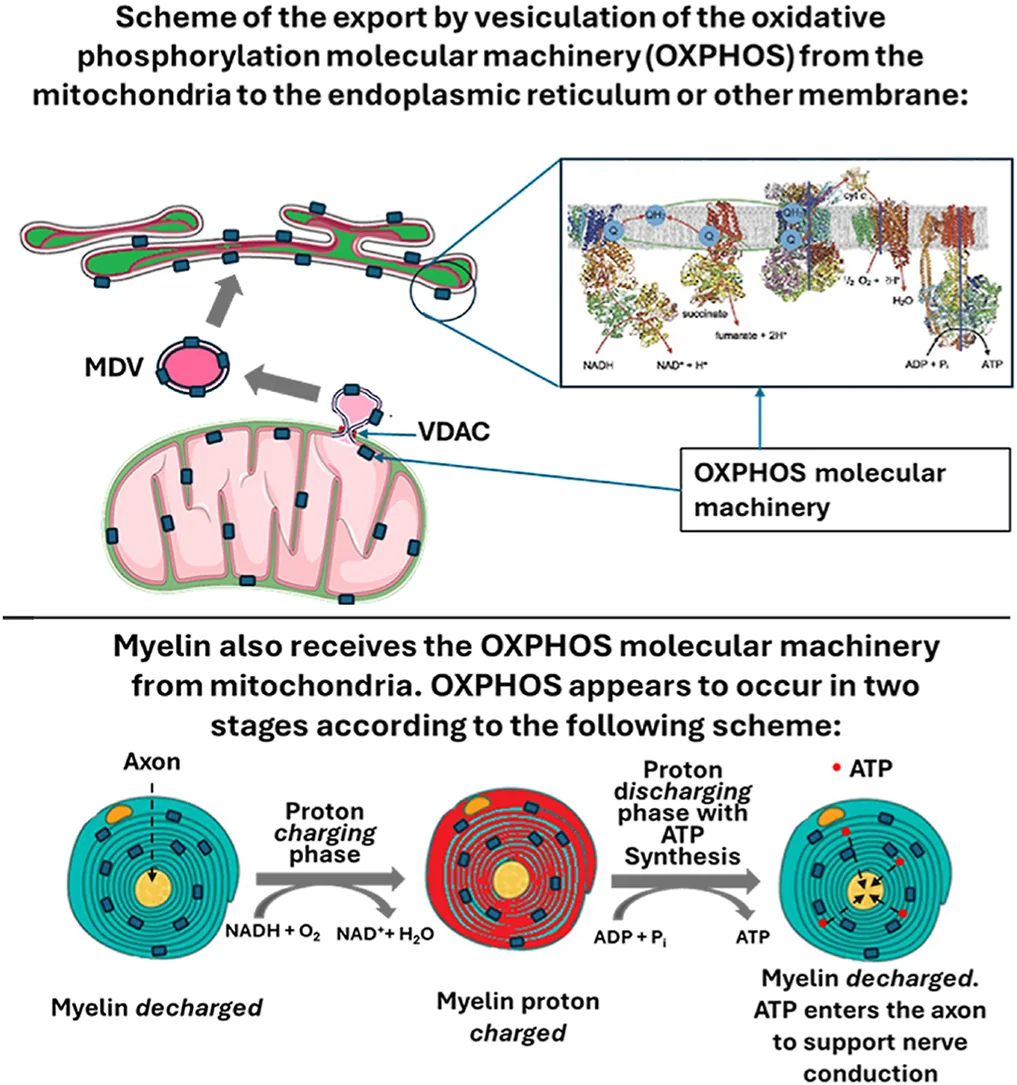

## 1 Introduction

After an oxygen-poor atmosphere the amount of oxygen raised enormously due to the oxygen production by photosynthetic bacteria during the Great Oxidation Event around 2.4–2.1 billion years ago [1]. So, to develop reactions that consumed oxygen, according to the endosymbiotic theory [2], the aerobic bacteria were phagocytized by ancestral eukaryotes, but this statement needs to be updated because it is unlikely that aerobic bacteria, precursors of mitochondria, were phagocytosed by an ancestral eukaryotic cell simply to supply it with ATP, a precious resource that would have helped these ancestral eukaryotes to better phagocytose these "intruders". Some have rightly called it the "*Suicide Hypothesis*” [3].

It has been hypothesized [4] that bacteria possessing OXPHOS, but only poorly developed internal membranes, acquired the capacity to expand their internal membrane surface (i.e., cristae) from ancestral eukaryotes [5], [6] as eukaryotes have highly developed endoplasmic reticulum.

This process, which leads to fusion of mitochondrial membranes with other cellular membranes, especially the endoplasmic reticulum, may have enabled OXPHOS functions to be expressed in other cellular membranes, a phenomenon referred to here as extra-mitochondrial ATP synthesis (EMAS) [7]. In fact, extra-mitochondrial OXPHOS has been reported in vertebrate photoreceptors [8], myelin [9], platelets [10], plasma cells [11], and sarcolemma of skeletal muscle [12]. This suggests that extramitochondrial oxidative phosphorylation (OXPHOS) is active in subcellular regions of most living organisms. Using an innovative FRET technique to measure ATP in HeLa cells, Imamura et al. [13] showed that extra-mitochondrial ATP concentration was almost twice that of mitochondrial ATP. This finding is confirmed by multiple reports measuring a cytosolic ATP concentration that exceeds the mitochondrial concentration [14], [15], [16], [17] albeit using different analytical techniques.

Furthermore, the chemiosmotic theory does not foresee mechanisms for energy storage, whereas such storage must be the basis of naturally occurring processes such as sleep. The present hypothesis, postulating the existence of "energetic stores by membrane proton buffers" satisfies this requirement and has already been applied to formulate a coherent and parameterized hypothesis on sleep [18]. This model provides estimates of the amount of energy that could accumulate during sleep in the form of protons associated with acid-base groups, including carboxylic and amino groups, in abundant myelin components such as proteolipid protein and myelin basic protein, energy that is converted into ATP to sustain the axonal nerve impulse during wakefulness [18], [19]. The membrane proton buffers appear as a ready-to-use energy storage system that has been detected in myelin and also in rod outer segments [20].

In developing this hypothesis, the principle that chemical processes are much more efficient when they occur in a homogeneous phase is taken into account. Therefore, proton movement is assumed to occur exclusively within the proteolipid phase of the membrane, as established by Hendrik Flegel et al. [21], whose findings confirm previous reports [22], [23], [24], [25], [26], [27]. A recent report further confirms that proton flows are entirely confined within the membrane [28]. It has also been proposed that the membrane could serve as an energy store for proton accumulation through its dissociable groups [29]. It should be noted that the recent experimental demonstration provided by Flegel et al. [21] puts to rest a debate that has lasted for more than half a century, establishing that FoF1-ATP synthase is driven by neither membrane potential nor proton-motive force. All these findings support the hypothesis formulated here. As discussed previously [7], this misunderstanding may arise from the erroneous interpretation of signals provided by so-called “membrane potential indicators”. These probes may reflect the degree of membrane protonation rather than membrane potential itself. Membrane potential may still exist, but it may be independently related to ATP synthesis.

This overall picture broadly reflects the chemiosmotic theory because it confirms the existence of proton movers, i.e., respiratory complexes I-IV, and ATP synthase as the machinery for ATP synthesis. The difference lies only in the coupling modalities, as protons move exclusively in the proteolipid phase of the membrane. Furthermore, this hypothesis predicts the proton capacitor function of the membrane, which is also exportable, and this complements the functions attributed to mitochondria as mediators of signal transduction and cellular processors [30].

This article is an adaptation of a previously published preprint [31].

## 2 Production of mitochondrial derived vesicles

A property of bacteria must be taken into account, namely their extreme versatility in producing vesicles [32], a property that has been retained by mitochondria, which possess a plurality of molecular devices for vesicle production, namely mitofusins 1 and 2, OPA1, and Drp1 [33].

Interestingly, their functional deficit causes serious neurological pathologies [34]. The production of mitochondria-derived vesicles (MDVs) in eukaryotes is a highly active and multifaceted process that has been the subject of extensive research in recent years [7], [33], [35], [36], [37], [38]. MDVs can fuse with the endoplasmic reticulum [39], where they form rafts containing approximately 24% mitochondrial proteins [40]. The myelin sheath also contains rafts [41], [42], which is consistent with the possible transfer of rafts to the oligodendrocyte endoplasmic reticulum (ER), where they would then fuse with the developing myelin sheath.

The study of MDVs thus opens up fascinating scenarios, particularly the visionary idea that mitovesicles bud from mitochondria to spread and fuse with intracellular membranes, primarily the endoplasmic reticulum, where ATP synthesis can occur *in situ* thanks to the import of the OXPHOS molecular machinery. Fig. 1 illustrates a possible vesiculation process of the mitochondrial inner membrane through the maxi-pore voltage-dependent anion channel (VDAC) [43], which is present in the mitochondrial outer membrane.

**Fig. 1:**
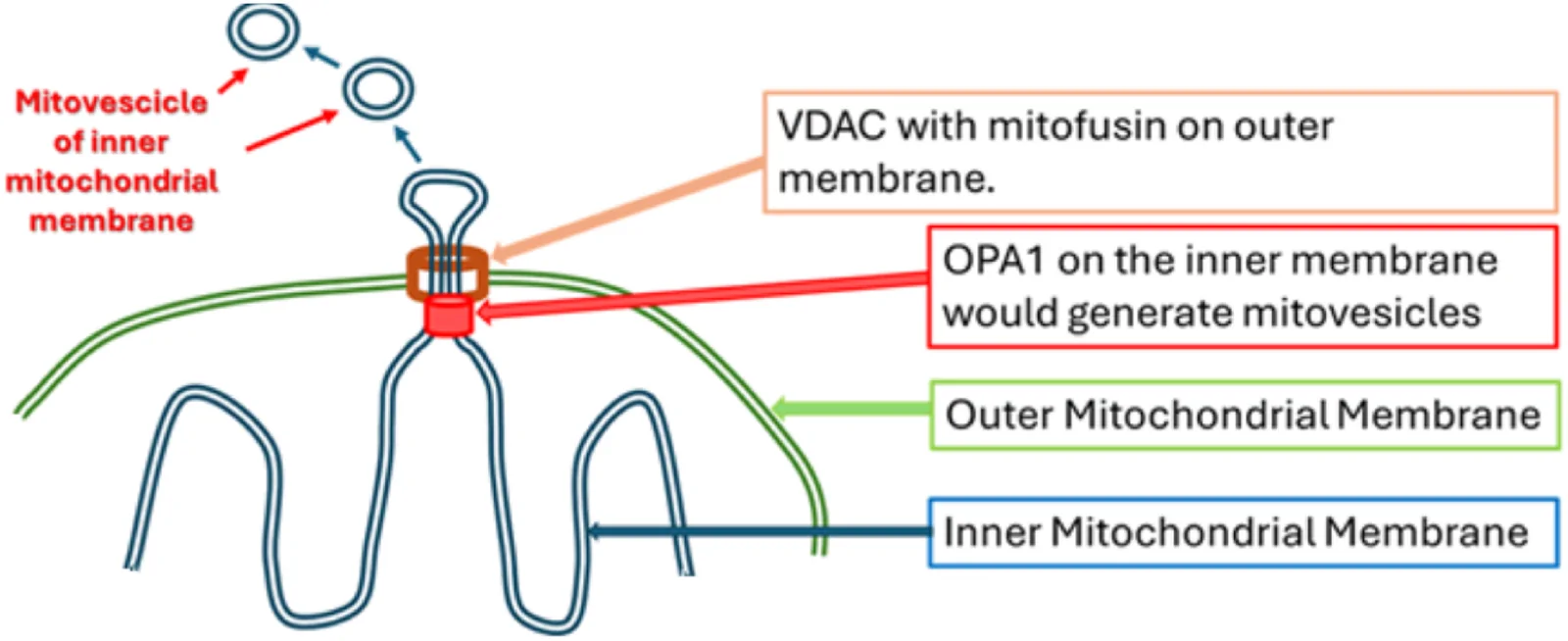
Proposed extrusion and delivery of mitovesicles derived from inner mitochondrial membrane fragments, with “narrowing” mediated by OPA1 inserted into the inner mitochondrial membrane and extrusion through VDAC inserted into the outer mitochondrial membrane.

The VDAC, which is located in the outer mitochondrial membrane, plays an important role, as it may extrude the inner mitochondrial membrane to produce MDVs. This hypothesis stems from the fact that VDAC is a protein consisting of 19 antiparallel β-strands arranged in a barrel shape, with an internal diameter of 3.15 nm [44]. However, VDAC may tetramerize [45], forming new hydrogen bonds between one end of one VDAC molecule and the end of another, thereby forming a ring with a diameter of approximately 13 nm. This structure could be suitable for “squeezing” and extruding MDVs, particularly with the action of Drp1, which have a single membrane bilayer derived from the inner mitochondrial membrane.

This vesiculation process would export the complex OXPHOS machinery to other sites, enabling ATP synthesis *in situ*.

All this goes beyond the traditional model, which attributes the aerobic synthesis of ATP exclusively to mitochondria. Notably, MDVs may contain FoF1-ATP synthase and synthesize ATP [46], [47], [48].

## 3 Formulation of a hypothesis on energetic stores by membrane proton buffers

Since the chemiosmotic theory [49] is not applicable to non-mitochondrial membranes expressing OXPHOS, it is necessary to integrate it with a hypothesis that also accounts for energy storage via membrane proton buffers. In doing so, previous reports addressing this topic have been taken into account [7], [50], [51], [52], [53], [54], [55], [56]. A recent article by S. Ji clearly identifies the limitations of the chemiosmotic theory [57]. Fig. 2A and B shows, respectively, the scheme predicted by the chemiosmotic theory and the essential features of the hypothesis formulated here.

**Fig. 2:**
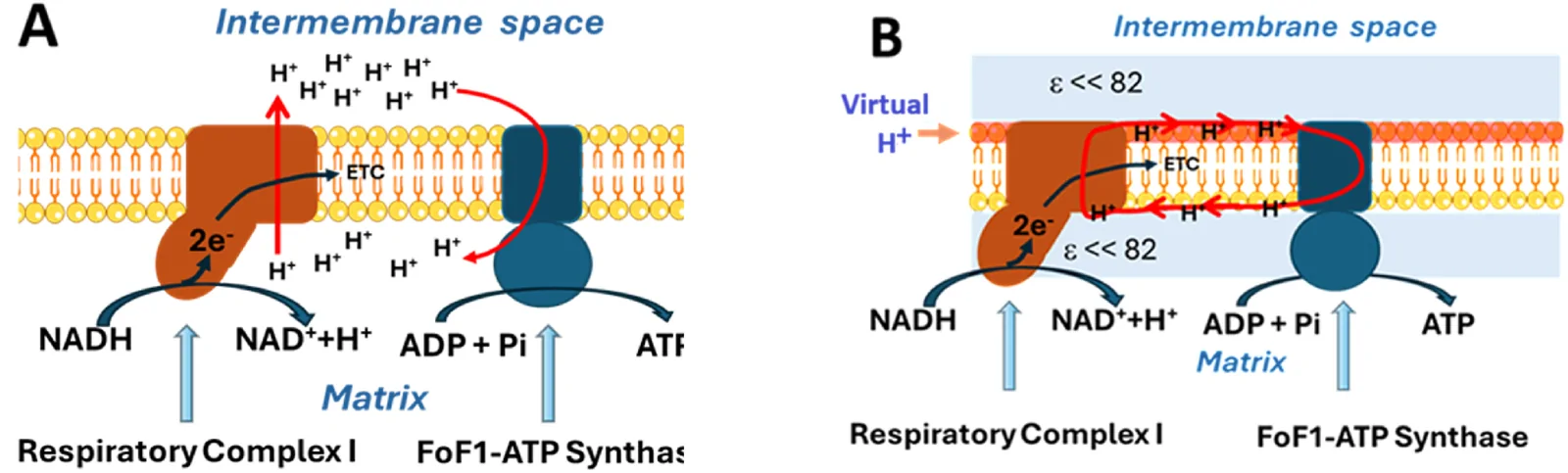
Only respiratory complex I is considered, as it oxidizes NADH by initiating the electron transport chain (ETC), coupled with the transfer of four H^+^ ions for each molecule of NADH oxidized. **A**: the protons accumulated in the intermembrane space—which would generate the proton-motive force—return to the matrix, rotating the F_0_ subunit, resulting in ATP synthesis. **B**: the existence of a layer with a dielectric constant ε ≪ 82 on both sides of the membrane confirm the insulating properties of the membrane relative to the aqueous bulk. The protons migrate along ionizable phosphate and nitrogen-containing residues of phospholipids in the intermembrane-space-facing hemilayer. They then pass through the Fo subunit of ATP synthase and return to the ionizable residues of phospholipids in the matrix-facing hemilayer.

For simplicity, only Respiratory Complex I is considered in Fig. 2A and B. This complex oxidizes NADH by initiating the electron transport chain (ETC), coupled with the transfer of four H^+^ ions for each molecule of NADH oxidized [58], [59], [60]. Fig. 2A illustrates the scheme of the canonical chemiosmotic theory: protons are transferred to the intermembrane space by Complex I, establishing the coupling with FoF1-ATP synthase. The subsequent return of protons to the matrix drives the rotation of the Fo subunit of the FoF1-ATP synthase nanomachine, which synthesizes ATP in the matrix.

This coupling, traditionally termed "delocalized coupling," is unrealistic, as the accumulation of protons in the intermembrane space is not possible; were it to occur, the mitochondrion would collapse due to excessive local acidity. Even Robert Williams, one of the staunchest opponents of the chemiosmotic theory, argued that protons could not accumulate within the intermembrane space [61], [62]. His famous objection was that protons transferred into the intermembrane space would be lost in a “Pacific Ocean” [63]. In support of this view, it has been pointed out that the number of protons in the microvolume of a mitochondrion can be very small [62].

It should be noted that, according to the chemiosmotic theory, protons drive FoF1-ATP synthase through the so-called membrane potential, or proton-motive force, which have been demonstrated to be equivalent in this context [64]. However, the novel aspect introduced here is that this movement depends on neither of these factors, as schematized in Fig. 2B.

In fact, in Fig. 2B, the hypothesis formulated here is schematized without either the proton-motive force or the membrane potential being represented. The protons moving from Complex I travel exclusively within the proteolipid membrane, in accordance with the principle stated above that processes must be distinguished according to the phase in which they occur. The proton current travels along the ionizable head groups of the phospholipids facing the intermembrane space without being released into it [21], [22], [65], [66], [67].

The protons are captured by the Fo subunit of the FoF1-ATP synthase nanomachine, which synthesizes ATP, and can return to Respiratory Complex I by traveling along the ionizable head groups of the phospholipids facing the matrix. These groups, which consist of phosphate and basic moieties such as ethanolamine and choline, can retain protons by accumulating them through Brønsted acid dissociation. This is the core of the hypothesis.

It is important to take into account that the inner mitochondrial membrane is isolated from the aqueous phase by a layer approximately 7–10 nm thick, with a dielectric constant ε < 82 [68], [69]. The “proton-motive force” exerted by Respiratory Complex I is difficult to formulate in thermodynamic and mathematical terms. This coupling can be traced back to localized coupling, which has already been proposed [70], [71], [72], [73] as an alternative to delocalized coupling and is based on the strict separation of processes occurring within the membrane from those occurring in the aqueous phase.

It should be noted that protons are never present in free form; rather, Grotthuss-type kinetic hopping occurs, involving the movement of the proton's positive charge but not its mass.

The requirement that protons never exist in a free state is also supported by evidence that biological membranes are permeable to protons [74], [75], [76], [77]. Accordingly, if protons were released into the aqueous phase during energy coupling, they would be expected to escape from the membrane and diffuse into the surrounding aqueous bulk. However, experimental evidence indicates that proton diffusion along the membrane surface is a preferential process relative to proton transfer into the bulk aqueous phase [27]. Furthermore, a recent and elegant experiment provides evidence supporting the hypothesis that protons are translocated within the membrane by proton-generating systems [21].

In conclusion, the traditional chemiosmotic theory has undergone a substantial conceptual modification in light of work carried out by several research groups, including the author and his own research team. According to this revised model, protons do not exist as free species in the water phase during energy coupling; instead, they move exclusively within the proteolipid phase of the membrane. Furthermore, the membrane itself can act as a proton capacitor, a property that can be enhanced in membrane systems containing proteins capable of contributing to this function. Myelin basic protein (MBP), which is present in great abundance within myelin, appears to play such a role, as discussed later.

## 4 Two phases of OXPHOS and the basics of the Membrane’s proton capacitor

Having established that proton translocation during oxidative phosphorylation (OXPHOS) occurs across the mitochondrial inner membrane [31], it is conceivable that the polar headgroups of membrane phospholipids may contribute to proton buffering [7]. Specifically, these groups could transiently retain protons during the oxidative phase of OXPHOS and subsequently release them during the ATP synthesis phase. This proposed mechanism, referred to here as the *Membrane Proton Buffer* function, would couple proton accumulation and release to the rotation of the Fo subunit of ATP synthase, thereby supporting ATP synthesis.

To conceptualize this mechanism, OXPHOS is considered to occur in two functionally distinct phases, an approach previously proposed in the context of sleep [18] and in models of the overall OXPHOS process, including the proposed existence of an intermediate energy accumulator [78]. Fig. 3A illustrates the Oxidative Phase, during which respiratory Complex I translocates protons from the matrix side of the inner mitochondrial membrane toward the intermembrane space. In the proposed model, proton transfer involves the polar headgroups of phospholipids on the matrix-facing and intermembrane-space-facing sides of the membrane. Fig. 3B illustrates the ATP Synthesis Phase, during which protons return toward the matrix through the Fo sector of ATP synthase, driving its rotation and enabling ATP synthesis. In this model, the polar headgroups of membrane phospholipids are proposed to contribute to proton retention and transfer along this pathway.

**Fig. 3:**
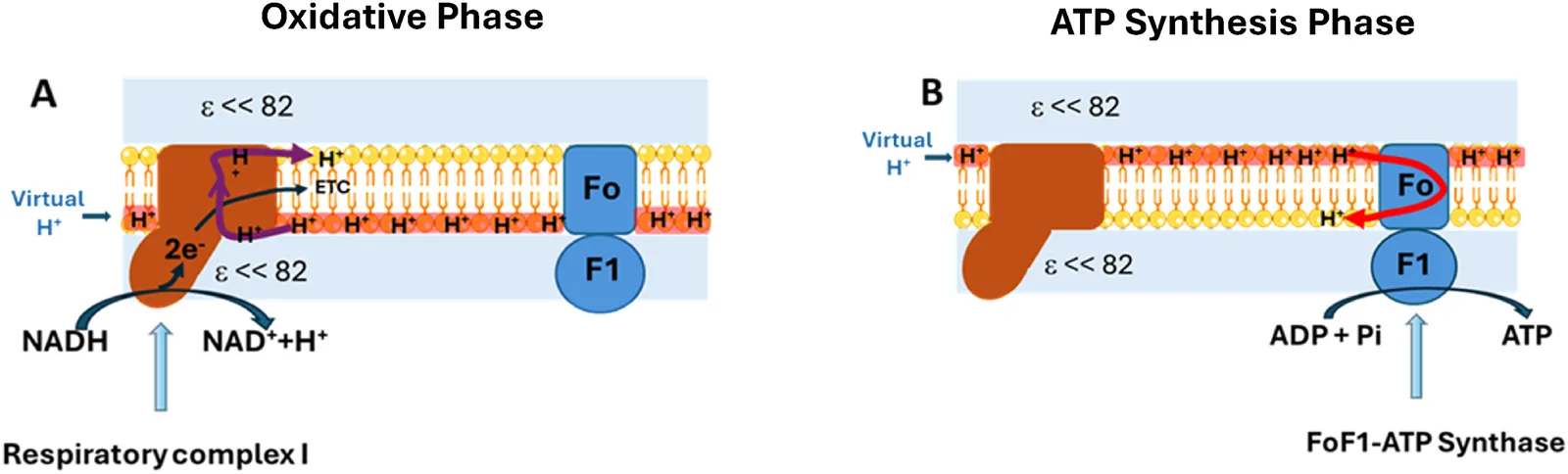
The diagram illustrates the phases that lead to the “charging” and “discharging” of the proton membrane buffer. For simplicity, only Respiratory Complex I is considered. This complex oxidizes NADH, initiating the electron transport chain (ETC), which is coupled with the transfer of four H^+^ ions for each molecule of NADH oxidized. **A**: is shown the “Oxidative Phase”: the protons are transferred from Complex I. **B:** is shown the proton transfer through the Fo subunit causing thw ATP synthesis in the matrix.

The proposed ATP Synthesis Phase operates when ADP and inorganic phosphate are available in the mitochondrial matrix. In their absence, ATP synthesis is limited, whereas respiratory proton pumping may continue to a degree determined by the prevailing membrane buffering capacity.

Within the proposed framework, this situation would favor the accumulation of protons in protonatable membrane groups, consistent with the hypothesized Membrane Proton Buffer function.

Notably, the proton-buffering capacity of the Membrane Proton Buffer can be enhanced by membrane proteins that act as proton buffers owing to their abundance of proton-dissociable groups [79]. Myelin is a prominent example, as its high content of myelin basic protein (MBP) and proteolipid protein (PLP) may substantially increase the proton-buffering capacity of the myelin sheath, as estimated in a preliminary calculation [18].

We reported measurements [20] supporting the absence of a proton capacitor in mitochondria, whereas the proton capacitor is operative in isolated myelin vesicles and rod outer segments. In Fig. 4, we describe the experiments in detail. The three preparations were incubated for progressively longer periods in Step 1 with the conventional respiratory substrates pyruvate + malate (PM). Isolated myelin (IM) accumulated protons on the myelin basic protein, thereby realizing the oxidative phase of OXPHOS.

**Fig. 4:**
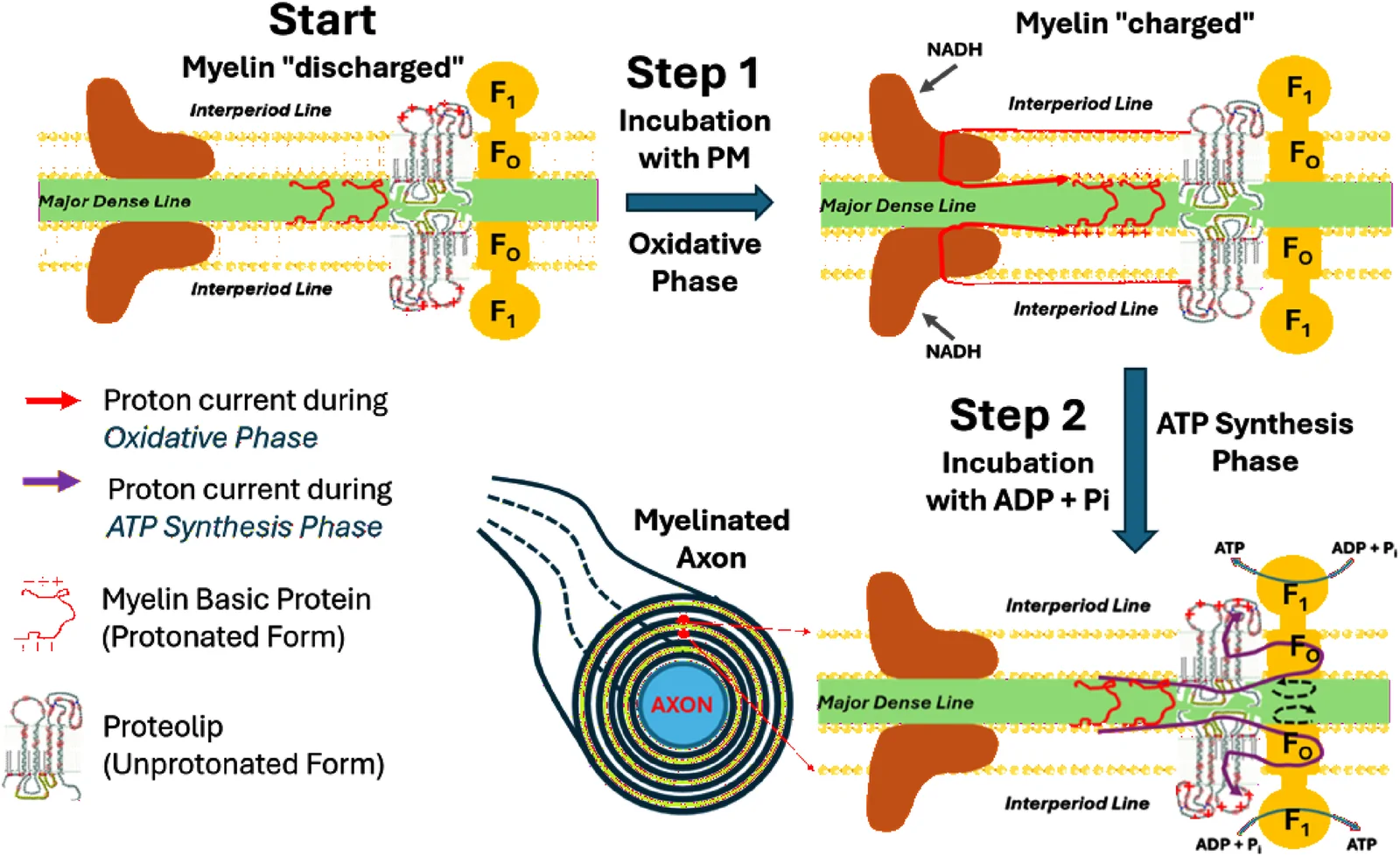
Explanation of the potential progression of the two phases of oxidative phosphorylation (OXPHOS)—the oxidative phase and the ATP synthesis phase—within isolated myelin (IM), which is capable of accumulating protons due to its high content of myelin basic protein and proteolipid protein. The experiment described in Fig. 4 of the article [20] is explained here. The starting material is “unloaded” IM, which accumulates protons in the proteolipid domain (PLP), facing the interperiod line. In Step 1, the IM is incubated for progressively longer periods with the respiratory substrates pyruvate/malate (PM). Respiratory complex I translocates protons from the PLP to the Myelin Basic Protein (MBP), located in the major dense line. The path of the protons is indicated by the red line. Step 1 represents the Oxidative Phase of OXPHOS. In Step 2, the samples incubated with PM are collected at successive time points, centrifuged to remove PM, and resuspended in ADP + Pi to complete the ATP Synthesis Phase.

The rationale is that, during incubation with PM, the first phase of OXPHOS (i.e., the oxidative phase; see Fig. 4) occurs, with oxygen consumption and proton charging of the membrane proton capacitor. The PM was then removed from the corpuscles by centrifugation. In the subsequent Step 2, the samples were resuspended in ADP + Pi. The ATP produced was then measured using the luciferin–luciferase method in a luminometer.

ATP is produced, thereby realizing the second phase of OXPHOS (i.e., the ATP synthesis phase). The amount of ATP produced reflects the energy stored during the first phase, and this amount increases with the duration of incubation of the myelin vesicles with PM. Mitochondria are completely incapable of storing energy [Fig. 4A of our article [20]].

The ATP synthesis phase requires the availability of ADP; in its absence, the myelin sheath is proposed to remain in a “charged” state. This interpretation is consistent with the sleep hypothesis [18], which proposes that gap junctions close during sleep in response to oleamide, thereby preventing ADP transfer from the axon to the myelin sheath. Upon awakening, oleamide is removed, allowing the gap junctions to reopen and ADP to become available for ATP synthesis. Consequently, ATP production can resume, enabling ATP to be supplied to the neuron during wakefulness.

Fig. 5 illustrates the proposed function of the membrane proton buffer in supporting the sleep–wake cycle. The sleep phase is proposed to correspond to the oxidative phase and is represented by the red line. The wake phase is proposed to correspond to the ATP synthesis phase and is represented by the purple line.”

**Fig. 5:**
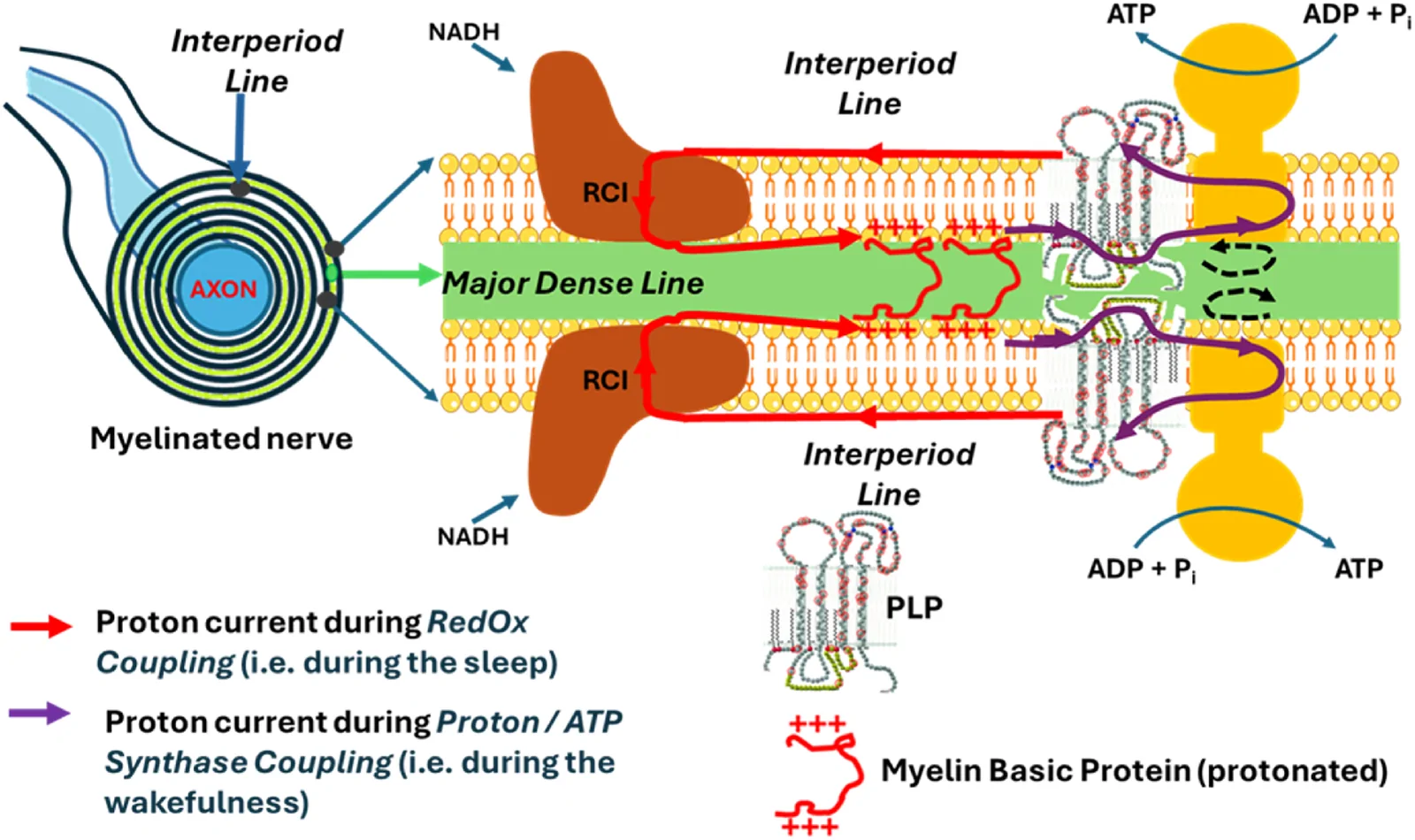
Scheme of the membrane proton buffer function in the myelin sheath to support the sleep–wake cycle. The myelin sheath surrounding the nerve is shown on the left, and a detail of the membrane arrangement is shown on the right. The interperiod line corresponds to the mitochondrial matrix, whereas the major dense line corresponds to the mitochondrial intermembrane space. **Red line =** Myelin “proton recharge” phase: proton current flows through the Fo subunit of ATP synthase, resulting in ATP synthesis by the F1 subunit immersed in the interperiod line.

## 5 Experimental work suggested to verify and test the hypothesis

Specifically, two phenomena are described here to complement the Chemiosmotic Theory [1]: the two-phase progression of OXPHOS and [2] the existence of the proton capacitor, which is dependent on the existence of the first phenomenon. Furthermore, everything proposed here is contingent on the fact that OXPHOS can also occur with high efficiency in extramitochondrial compartments.

The experiments that can be proposed, based also on the information provided here, should primarily be performed on myelin isolated in the laboratory according to the widely used procedure of Norton and Poduslo [80]. Briefly, isolated myelin can be incubated with the respiratory substrates pyruvate and malate (PM) to induce the oxidative phase of OXPHOS. PM is then removed by centrifugation, and ADP + Pi is added to induce the ATP synthesis phase of OXPHOS. The rationale for this experiment is illustrated in § 4.

Using the same approach, other preparations can be analyzed to investigate the possible existence of the proton capacitor. Skeletal muscle and kidney are tissues that may possess rapid energy-storage capabilities, as extramitochondrial OXPHOS has already been identified in skeletal muscle [12], along with a proton buffer [81].

In podocytes, on the other hand, which are key cells in the kidney responsible for filtering the circulating blood and have a very high energy demand—higher, on a weight basis, than that of oligodendrocytes—high OXPHOS activity has been identified without a corresponding high production of mitochondria [82]. The kidney may therefore be a tissue in which elevated extramitochondrial OXPHOS activity, associated with a proton capacitor, is present.

## 6 Conclusions and perspectives

The traditional statement that *“mitochondria are the powerhouses of the cell”* can be expanded to suggest that *“mitochondria are exporters of the molecular machinery required for energy production.”* Mitochondria assemble numerous proteins involved in oxidative phosphorylation (OXPHOS), which, according to the proposed model, may be transported in vesicles to other intracellular and extracellular locations, where they could potentially support autonomous ATP synthesis.

OXPHOS can be conceptually divided into two phases: an *oxidative phase*, during which electron transport drives proton translocation and the establishment of accumulation of protons within the membrane-resident proton buffering system, and an *ATP synthesis phase*, during which the return of protons through ATP synthase drives ATP production. The first phase may also contribute to proton accumulation by dissociable chemical groups, particularly those associated with the polar headgroups of phospholipids. However, mitochondria may have a limited capacity to store protons in such chemical reservoirs. In this context, the proposed export of OXPHOS machinery could allow these components to relocate to cellular compartments where other molecular constituents might increase their effective proton-storage capacity. Myelin and the outer segments of rod photoreceptor cells have been suggested as possible examples of such environments [20].

All this requires that the chemiosmotic theory be updated [56], [83], [84], [85], [86], [87], [88], [89]. Accordingly, the hypothesis of energetic storage by membrane proton buffers has now been formulated. This hypothesis posits a clear distinction between processes occurring within the membrane and those taking place in the aqueous phase that bathes both sides of the inner mitochondrial membrane, or other cellular membranes to which OXPHOS is exported. Extramitochondrial OXPHOS has been found in the plasma membrane [90], [91], [92], [93], [94], [95], [96], the endoplasmic reticulum [97], [98], the sarcoplasmic reticulum [12], and the widely distributed mitochondrial-derived vesicles (MDVs) [35], [46], [47], [99].

The presence of macromolecules and enzymatic activities characteristic of oxidative phosphorylation (OXPHOS) in 703 cubes (3 mm per side) obtained by voxelizing a frozen human brain [100] can be interpreted, as argued here, as evidence of widespread extramitochondrial OXPHOS across various brain regions. This activity appears to occur at levels exceeding those estimated on the basis of the density of morphologically identifiable mitochondria.

This new approach addresses some of the inconsistencies of the original chemiosmotic theory by providing a simplified interpretation in which (i) the processes involved occur predominantly within the proteolipid phase of the membrane, and (ii) changes in the fluorescence signals produced by so-called *membrane potential indicators* are interpreted differently. Specifically, these indicators may reflect the protonation state of the membrane itself rather than its electrical potential, as previously suggested [7]. Furthermore, it has been reported that ATP synthesis by the FoF1-ATP synthase is independent of the membrane potential [101], [102].

Surprisingly, some of the properties highlighted here have already been demonstrated experimentally by Russian scientists half a century ago. In 1975, it was shown that an aqueous buffer dispersion of the hydrocarbon octane, incorporating the weak hydrophobic acid dinitrophenol (DNP), was able to accumulate protons transferred into it through the action of bacteriorhodopsin upon illumination [103].

The following year (1976), it was demonstrated that the octane/DNP suspension, which had been “charged” with protons during a previous phase by bacteriorhodopsin, according to the methodology described in the 1975 study, was able to synthesize ATP when ADP + Pi and FoF1-ATP synthase were added to the suspension [104].

Years later, we can say that OXPHOS had already been established in two phases: in 1975, the oxidative phase, involving proton loading of the hydrophobic octane phase containing DNP, was demonstrated, while in 1976, the ATP synthesis phase was demonstrated.

Also relevant are the Russian scientists’ precise demonstrations of direct proton transfer between the respirasome and FoF1-ATP synthase [25], the possibility of storing energy in protons incorporated by ionizable membrane groups [29], and the modes of proton coupling between respiratory complexes and FoF1-ATP synthase [26].

The import–export processes associated with the OXPHOS molecular machinery have recently been discussed in the article *“Mitochondria-derived vesicles: potential nano-batteries to recharge the cellular powerhouse”* [36]. The concept of vesicles as potential cellular “batteries” is reminiscent of the proposed membrane proton buffer, which now is described as a central component in the accumulation and storage of energy in the form of protons. According to this hypothesis, protons are incorporated into membrane-associated acid–base buffers, potentially providing a mechanism for energy storage that may contribute to a unified theory of sleep [18]. Furthermore, the proposed proton-buffering capacity of biological membranes has been linked to the formation of inverted micelles, which may increase their capacity to store protons [87].

The capacity of membranes to store protons and, therefore, energy, increases with the developed surface area of the membrane. This appears to be an evolutionary advantage acquired by mitochondria, which have developed an internal membrane folded into cristae, unlike the bacteria from which they derive, thereby increasing their surface area.

Interestingly, the sleep hypothesis requires OXPHOS to function in a manner consistent with the new hypothesis on membrane proton buffers as energy stores formulated here. This finding may pave the way for understanding energy homeostasis in energy-intensive organs, such as the heart, skeletal muscle, and kidneys, where extramitochondrial OXPHOS appears to be highly active. Finally, the existence of membrane proton buffers in extracellular compartments, as already demonstrated in myelin and in the outer segments of rod photoreceptors [20], could complete the framework according to which mitochondrial function constitutes a cellular processor [30] that regulates energy homeostasis [105] among proton buffers plausibly distributed across various subcellular compartments. At a glance, a close correlation emerges between mitochondrial function and memory [106], [107], and thus with energy homeostasis, in which membrane proton buffers appear to play a pivotal role.

It has been demonstrated in mice that restoring proper mitochondrial function allows for the restoration of memory [108]. Given that sleep is known to reorganize memory [109], the link between mitochondrial function of myelin, memory, and sleep is becoming increasingly substantiated.

## Declaration of competing interest

The author has no conflict of interest to declare.

## Data Availability

No data was used for the research described in the article.
